# Splenic Abscess: A Rare Complication of the UVC in Newborn

**DOI:** 10.1155/2014/903421

**Published:** 2014-01-08

**Authors:** Ameer Aslam, Emad Sadek Ahmed Shatla, Sameera Imanullah, Elsaid M. A. Bedair

**Affiliations:** Department of Pediatrics, Pediatric Emergency Centre, Hamad Medical Corporation, P.O. Box 3050, Doha, Qatar

## Abstract

Splenic abscess is one of the rarest complications of the UVC in a newborn and it is hypothesized that it could be due to an infection or trauma caused by a catheter. The case that is being reported presented with abdominal distension and recurrent desaturation with suspicion of neonatal sepsis versus necrotizing enterocolitis. However, the final diagnosis was splenic abscess as a complication of an inappropriate UVC insertion which was discovered by abdominal ultrasound. The patient was given broad spectrum antibiotics empirically and the symptoms were resolved without any surgical intervention. Such cases and controlled studies need to be reported in order to identify further causes and risk factors associated with splenic abscess in a patient with UVC which can eventually help us adopt preventive strategies to avoid such complications.

## 1. Introduction

Common complications of UVCs in a newborn are infection, hemorrhage, vessel perforation, creation of a false luminal tract [1], hepatic abscess or necrosis [2], air embolism, catheter tip embolism, portal venous thrombosis [3], dysrhythmia, and pericardial tamponade or perforation. Splenic abscess can occur in a newborn without any significant symptom and can resolve without any significant intervention but it could per se be the fatal and lethal complication in patients with UVC. Splenic abscess usually is caused by hematogenous embolization and contagious spread. As saving the central lines in newborn is the most common and integral part of any NICU for treatments and nutrition, but care should be taken to avoid the morbidity and mortality related to UVC.

## 2. Case Report

A Sudanese female neonate born at 25 weeks of gestation with birth weight of 980 grams whose mother was known to have autoimmune hepatitis and primary biliary cirrhosis. The patient developed respiratory distress at birth which needed endotracheal intubation connected to mechanical ventilator after giving surfactant followed by UVC and UAC insertion. She was started on Ampicillin and Amikacin after sepsis screen. Moreover, caffeine citrate and total parental nutrition were started from the first day of life. Initial chest X-ray showed ETT in situ, bilateral hazy lung field but normal abdominal gas pattern. Next day, she developed jaundice without any set up which resolved with phototherapy in 2 days. Cranial ultrasound was reported as normal while insignificant PDA was reported by echocardiography.

**Figure 1:**
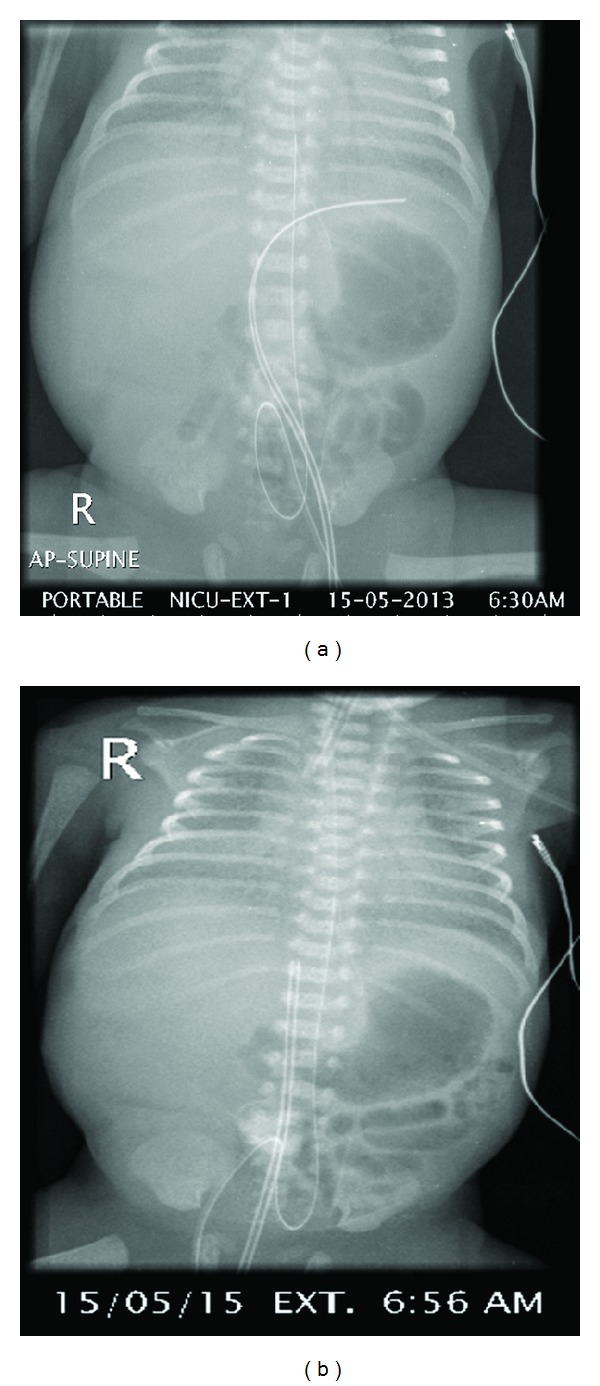
Plain X-ray of the abdomen demonstrating the UVC directed to the left within the splenic vein (a) which is corrected after 26 min (b).

**Figure 2:**
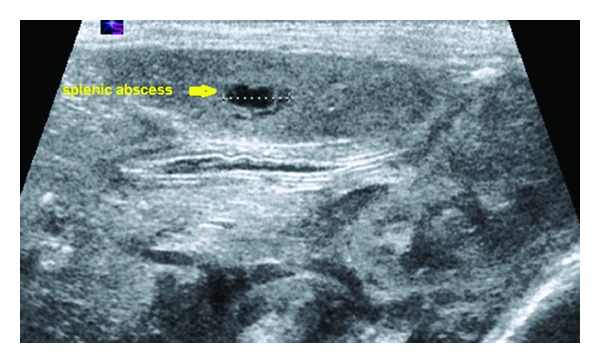
US examination demonstrating solitary small well-defined splenic abscess.

Abdominal X-ray to localize the position of the UVC showed it to be directed to the left side in splenic vein which was corrected immediately ( Figures 1(a) and 1(b)). However, abdominal X-ray done on the 5th day of life showed gas in the portal vein. Abdominal ultrasound revealed intrahepatic gas at portal venules (Figure 2) and slight hepatosplenomegaly. The splenic parenchyma showed two focal lesions measuring 13 mm and 5 mm with central liquefactions, suggesting abscess and splenic vein was seen patent. Spleen was palpable clinically from the 6th day of life. Pediatric surgical and infectious diseases teams were involved. Abdominal MRI was recommended by the pediatric surgical team while infectious diseases team recommended changing antibiotics to Tazocin, Teicoplanin, and fluconazole. MRI done showed no splenic focal lesions with an impression of sonographically located lesions probably beyond the resolution of the MRI. Patient had blood with ETT suction and CXR was showing lung opacity. UVC was removed on the 7th day of life. Next day patient developed symptoms of PDA that needed intravenous Ibuprofen, hence, repeated echocardiography after 5 days showed closed PDA. Culture of the sterile fluids (urine, blood, and CSF) showed no growth and C-reactive protein less than 5 mg/dL. Patient was started on tropic feeds on 9th day of life. Nevertheless, splenic abscess did not require any surgical drainage.

## 3. Discussion

Venous cannulation has been in regular use in neonates since the 1940s [4]. Umbilical catheterization is the most anxiety-creating skill in the neonatal intensive care unit because of neonatologists' concerns towards complications of UVCs. The well-known complications of UVCs are infection, hemorrhage, vessel perforation, creation of a false luminal tract [1], hepatic abscess or necrosis [2], air embolism, catheter tip embolism, portal venous thrombosis [3], dysrhythmia, and pericardial tamponade or perforation (if the catheter is advanced to the heart) [5–7].

Splenic abscess per se is a rare entity; so far reported cases are 600 from the international literature [8], which may occur due to the hematogenous embolization or contiguous spread. The former is embolization either to the normal spleen in case of septic endocarditis, IV drug abusers, and immune compromised situation or to a previously altered splenic architecture in case of infracted spleen due to sickle cell disease, vasculitis, and trauma. The latter may result from the direct involvement from a pancreatic abscess, bowel perforation, or subphrenic abscess [9, 10]. *Staphylococcus* and *Streptococcus* are the common pathogens. Imaging by common abdominal X-ray or ultrasound may be suggestive, but the lesion is usually revealed via computed tomography (CT).

The splenic abscess in the above-reported case was managed medically without any surgical intervention; therefore the direct isolation of the pathogen from the abscess itself was not performed. Due to the seriousness of the potential implications of the splenic abscess, including a threat to life itself, the most usual treatment currently applied is splenectomy, followed by rapid clinical improvement, which is not a case in this patient. It is also interesting to know that abdominal ultrasound can detect splenic abscess which can be missed by MRI that is what occurred in the above case.

After the most common cause which is infection, the clinical picture of this child signifies that splenic abscess due to complication of UVC might be due to trauma. But the evidence from multiple case reports suggests that cardiac tamponade is one of the fatal complications if the catheter is not placed properly as the tip of the catheter can easily traumatize the pericardium. So it could be hypothized that splenic abscess could be the complication either because of infection from the UVCs or due to improperly placed catheter which might have traumatized the spleen. Although this case did not show any fatal complication it might have happened if early intervention was not done in timely fashion.

## 4. Conclusion

In future, such cases and controlled studies need to be reported in order to identify further causes and risk factors associated with splenic abscess in a patient with UVC which can eventually help us adopt preventive strategies to avoid such complications. This complication might be considered in every newborn with central venous access who presents with hepatosplenomegaly.
